# The organization of healthcare work in the light of ergology: experiences in the COVID-19 pandemic

**DOI:** 10.1590/1980-220X-REEUSP-2022-0261en

**Published:** 2023-04-28

**Authors:** Wagner Ferreira Monteiro, Darlisom Sousa Ferreira, Kássia Janara Veras Lima, Igor Castro Tavares, Flávia Regina Souza Ramos

**Affiliations:** 1 Universidade do Estado do Amazonas, Fundação de Medicina Tropical, Pós-graduação em Medicina Tropical, Manaus, AM, Brazil.; 2 Universidade do Estado do Amazonas, Programa de Pós-graduação em Enfermagem em Saúde Pública, Manaus, AM, Brazil.; 3 Universidade Federal de Santa Catarina, Programa de Pós-graduação em Enfermagem, Florianópolis, SC, Brazil.

**Keywords:** Work, Health Personnel, COVID-19, Health Services, Trabajo, Personal de Salud, COVID-19, Servicios de Salud, Trabalho, Pessoal de Saúde, COVID-19, Serviços de Saúde

## Abstract

**Objective:**

To understand the work organization of health professionals when coping with the COVID-19 pandemic in Manaus.

**Method:**

This is a qualitative case study which adopted ergology as theoretical framework. Data production used document analysis and semi-structured interviews with 33 health workers from the Health Care Network. The resources of the software Atlas.ti 8.0 were used for data analysis.

**Results:**

The precepts of Thematic Networks analysis revealed the following categories: “Reordering services and functions”; “Incorporation and management of instruments application”; “Professional experiences and tactics: changing roles, attitudes and relationships”.

**Conclusion:**

It was found that they express a dynamic view of the organizational process, in which the worker, when discussing past standards and comparing his/her knowledge, experiences and values, modifies the environment, flows and conducts as needed, facing the lack of safety, conditions and solidity of the technical bases of work.

## RESUMO

**Objetivo:**

O estudo buscou compreender a organização do trabalho de profissionais de saúde no enfrentamento da pandemia de COVID-19 em Manaus.

**Método:**

Trata-se de uma pesquisa qualitativa, do tipo estudo de caso, que adotou como referencial teórico a ergologia. A produção dos dados empregou análise documental e entrevista semiestruturada com 33 trabalhadores da saúde da Rede de Atenção à Saúde. Utilizaram-se os recursos do software Atlas.ti 8.0 para análise de dados.

**Resultados:**

Os preceitos da análise de Redes Temáticas revelaram as seguintes categorias: “Reordenamento de serviços e funções”; “Incorporação e gestão da aplicação de instrumentos”; “Experiências e táticas profissionais: mudanças de papeis, atitudes e relações”.

**Conclusão:**

Constatou-se que eles expressam uma visão dinâmica do processo organizacional, em que o trabalhador, ao discutir normas do passado e comparar seus conhecimentos, experiências e valores, modifica o ambiente, os fluxos e as condutas conforme a necessidade, enfrentando a falta de segurança, de condições e de solidez das bases técnicas do trabalho.

## RESUMEN

**Objetivo:**

El estudio buscó comprender la organización del trabajo de profesionales de salud en el enfrentamiento de la pandemia de COVID-19 en la ciudad de Manaus, Brasil.

**Método:**

Se trata de una investigación cualitativa, del tipo estudio de caso, que adoptó el referencial teórico la ergología. La producción de los datos empleó análisis documental y entrevista semiestructurada con 33 trabajadores de salud de la Red de Atención a la Salud. Fueron utilizados los recursos del software Atlas.ti 8.0 para analizar los datos.

**Resultados:**

Los preceptos del análisis de Redes Temáticas revelaron las siguientes categorías: “Reordenamiento de servicios y funciones”; “Incorporación y gestión de la aplicación de instrumentos”; “Experiencias y tácticas profesionales: cambios de roles, actitudes y relaciones”.

**Conclusión:**

Se constató que ellos expresan una visión dinámica del proceso organizacional, en la que el trabajador, al debatir normas del pasado y comparar sus conocimientos, experiencias y valores, cambia el ambiente, los flujos y las conductas de acuerdo a la necesidad, enfrentando la falta de seguridad, de condiciones y de solidez de las bases técnicas del trabajo.

## INTRODUCTION

In recent years, the emergence of new infectious diseases has had repercussions beyond the cases and deaths themselves, as it triggers several questions about the attribution of national public health systems of legitimating its surveillance and health care, regarding the opportunity for prior identification and the power to contain new outbreaks^(1)^. In Brazil, an inter-ministerial working group was set up and the surveillance of human infection by the new coronavirus was gradually built as new technical and scientific evidence was published, conceiving actions of notification, registration, investigation, management, and adoption of preventive measures^(2)^.

Studies highlight the serious impacts on health professionals’ mental health in different countries, related to depression, anxiety, stress, insomnia and fear, among others, requiring coping alternatives, psychological support, and new strategies to promote resilience^(3)^. The causes of various disturbances and symptoms stem from the lack of workers, physical exhaustion when caring for an increasing number of patients, co-workers’ health problems, insufficient adequate support and personal protective equipment^(4,5)^. Therefore, there is a need to energetically prepare and structure health services to avoid damage to services and prevent still imponderable future consequences for workers^(6)^.

To understand these human work dynamics, studies in the field of ergology, a theoretical-methodological perspective to understand and transform work, stand out^(7)^. Ergology is a multidisciplinary approach to analyze work as an activity, considering a mix of technical and human behavior aspects that are essential for its understanding. It proposes discussing the world of work and organizations from the point of view of multiple knowledge and experiences, as it recognizes the different singularities and gaps between prescribed work and real work, in permanent recreation and production of reserves of organizational, social and political alternatives^(8)^. Its framework has been useful in studies involving the work process in health, either as a method or theory, with the aim of better understanding this work complexity^(9)^.

Ergology seeks to understand work situations, including the way they are regulated through normative and prescriptive sets of technical-scientific, financial, or organizational nature, which generate acts, behaviors, and technological uses. The purpose of this combination of concepts and standards is to predict any activity that workers will perform in the organizational environment. In ergology, the so-called antecedent standards, which guide the work in a never absolute way, articulate the set of regulations to cultural, historical, and value aspects^(9)^.

During his/her performance and taking unforeseen events into account, the agent renormalizes actions and tasks, recreates and remodels his/her professional trajectory, and invests in his/her knowledge in a personal, unique, and singular way, imprinting his/her identity to the task, in a continuous process of resingularization or redefinition of the activity itself. The comprehension of this process is important for carrying out actions and for the debate situated by the recognition of the inevitable renormalization of the activity and the real working condition^(10,11)^.

Therefore, it can be stated that all activities are, in themselves, problematic, and carry a drama for the worker, as they represent tension between possible choices – the dramatic use he will make of himself^(10)^. The drama of use of the self is connected to the daily tensions/dramas that permeate work, in a scenario inhabited by different stories or subjects – the place of confrontation and variability where the debate of standards that will carry out and establish its choices and arbitrations of values^(12)^ takes place.

Recognizing the productivity of the ergological perspective in the analysis of work and, in particular, its potential contribution to understanding the multifaceted context of crisis that has characterized health work during the COVID-19 pandemic, the study sought to understand the organization of the work of health professionals in coping with the COVID-19 pandemic based on concepts of the ergological approach. In addition, the study scenario, represented by one of the important epicenters of the pandemic in Brazil and in the world, demands critical knowledge, in what regards combining the description of the crisis situation of the system with the alternatives built in the transformed and transforming health workers’ experiences.

## METHODS

### Design of Study

This is a qualitative case study, which integrates a macro study that deals with the experiences built during coping of the COVID-19 pandemic in Manaus, Amazonas, Brazil.

### Population

A total of 33 health workers participated in the study. Among these, 13 nurses, 7 physicians, 6 nursing technicians, 2 community health agents, 2 dentists, 1 physiotherapist, 1 oral health assistant, and 1 social worker. The magnitude of the data (number of similar findings) confirmed the saturation of the data, or the density of the interpretations obtained, with no need for descriptive additions about the object of study^(13)^.

### Local

The study scenario was the Health Care Network for facing COVID-19 in Manaus-AM, among these, 4 Basic Health Units, 03 Hospitals, 1 Field Hospital, 1 Emergency Care Unit, and 1 Emergency Care Service. These services were chosen due to the fact that they are a priority gateway for the care of patients suspected of being infected with the new Coronavirus.

### Selection Criteria

Health workers linked to services involved in coping with COVID-19 were included in the study, taking the representation of different health services into account and using as an inclusion criterion to be working in the service mentioned for at least 01 month.

### Data Collection

Data production took place in the second half of 2020 using a semi-structured interview and document analysis. The interviews were previously scheduled, carried out at the workplace, by the main researcher or a duly trained collaborator, using a previously prepared script, containing guiding questions about the work process in coping with the COVID-19 pandemic, including focus on organizational arrangements and ways to work, developed strategies and experiences in face of standards and (individual and team) contingencies. The interviews lasted an average of 45 minutes, were recorded (audio) and later fully transcribed by the researcher.

### Data Analysis and Treatment

The analytical process used resources from the Atlas.ti 8.0 software (*Qualitative Research and Solutions*)^(14)^ and propositions of Thematic Network Analysis, consistent with the adopted theoretical framework. A thematic network is presented as a sensitive tool or technique for the systematization and presentation of qualitative data analysis; it shares the actions of discovering themes at different levels and exploring the understanding of an issue or the meaning of a conception with the thematic analysis, adding resources for structuring and representing these themes^(15)^.

The software allowed the creation of a hermeneutic unit for the treatment of data from the pre-analysis, after the interviews were transcribed and revised. Then, the thematic network analysis process consisted of phases and steps, which start from the breakdown of the text into explicit groundings and implicit meanings, originating, through this process, the thematic network Organization of Work.

### Ethical Aspects

Throughout the investigation process, the rules of ethics in research involving human beings and the indications of Resolution no. 466/2012^(16)^, of the National Health Council, were followed. Consent was obtained from the State and Municipal Health Departments and the study was approved by the Research Ethics Committee of the Universidade do Estado do Amazonas under Opinion No. 4.085.240 of 2020. All participants were duly informed about the research, signed the Free and Informed Consent Form and had their rights guaranteed. To preserve secrecy and anonymity, the fragments of the participants’ statements were identified by a code – the letters TS, of health workers (*trabalhadores da saúde* in Portuguese) followed by the participant’s serial number (TS01, TS02…).

## RESULTS

**Figure 1.**
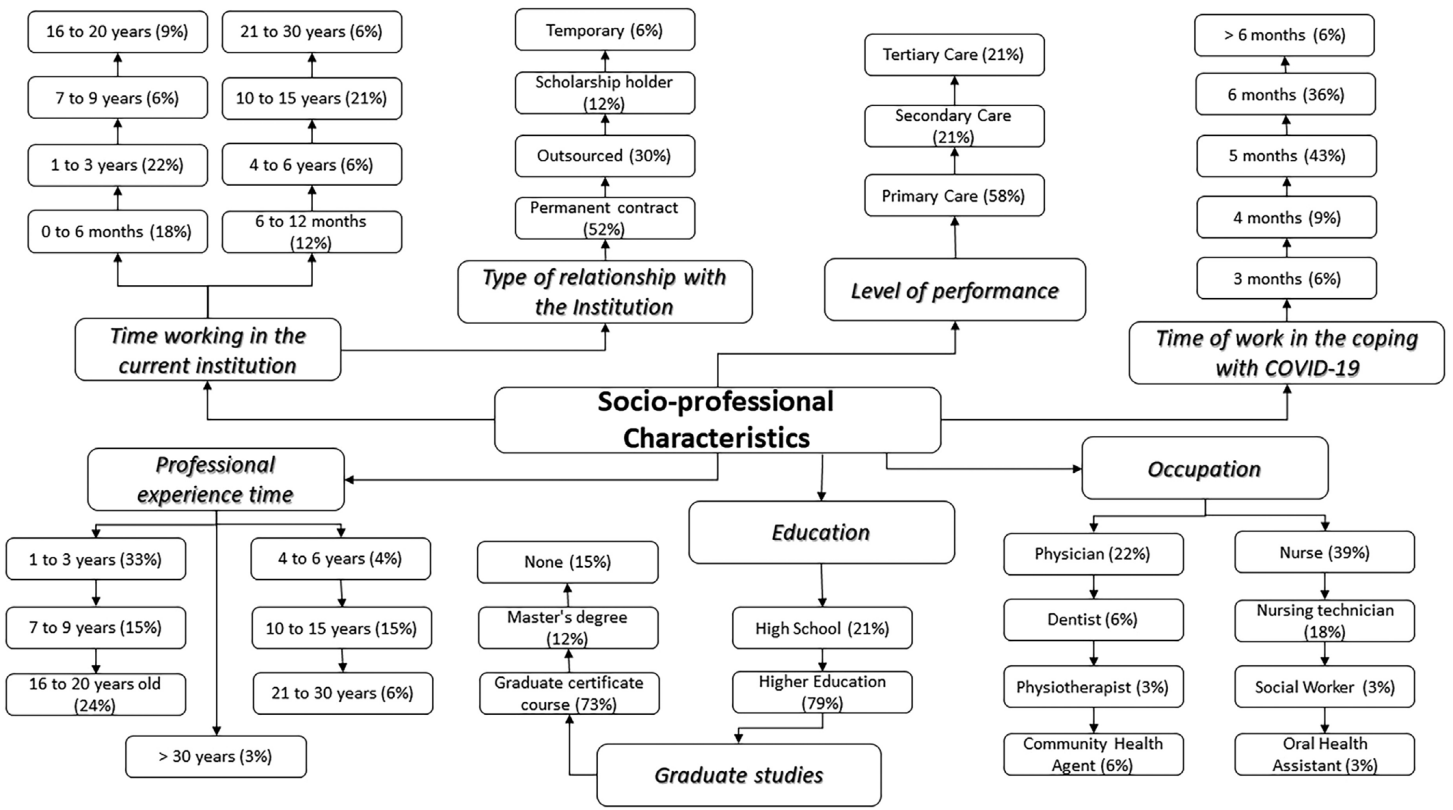
Socio-professional profile of health workers from the Health Care Network coping with COVID-19 – Manaus, AM, 2020.

Participants were predominantly women, young adults, with significant experience (63% with more than 7 years of experience), on a permanent contract basis (52%), and with a graduate degree (85%) (Figure 1).

**Figure 2.**
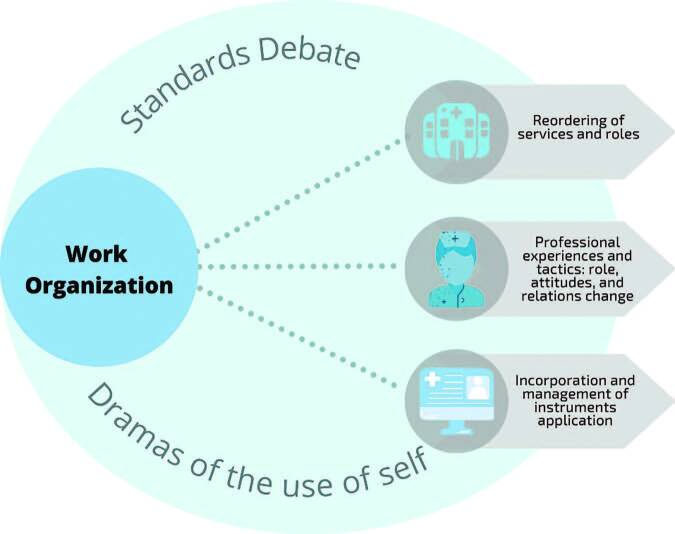
Work Organization Thematic Network.

The study results were structured based on the following themes: 1) “Reordering services and functions”; 2) “Incorporation and management of instruments application”; 3) “Professional experiences and tactics: changing roles, attitudes and relationships”, as seen in Figure 2. The three themes represent work challenges and demands that motivated the debate of standards between a prescription already assimilated to the teams’ ways of doing things, new prescriptions in constant change, and the emergent dramas of self-use in and through work.

### Reordering Services and Functions

One of the issues that the COVID-19 pandemic inserted into the debate was the need to rethink the way health work is carried out in the context of both the services provided by health units and the roles played by workers.

For professionals to adapt to the new global context of the pandemic, health services have undergone and are undergoing institutional reorganization, for the organization and reorganization of actions provided to the population.

*The first step was having to adapt, the second step was adapting the patients’ characteristics and the characteristics of the care itself, because we were treating chronic patients (…). And we started to see people with acute respiratory conditions and also waiting for very immediate answers; we were working with minimally objective criteria, but having to deal with the subjectivity of the thing and the other problems that kept happening. (TS25)*

*When the pandemic broke out, it totally changed our routine, our workflow. Our urgency/emergency worked in a different way, in a different flow, when the pandemic broke out there was isolation, all the wards that supported the resuscitation room were all for COVID. (TS04)*

*We stayed at the doors, doing it in a way so that there was no proliferation from the screening and preparation of the patient who came with the flu syndrome (…) we took it from the Ministry, from the management and implemented it in the unit. Nurses, technicians, even the management team itself helped, we were all on one hand, everyone contributed, social workers and community health agents. (TS09)*

After the transformation of these services, each environment determined its priorities at the time of service, creating ways to deal with the scenario imposed by the pandemic and to reorganize its work practices.

*First, there was the issue of challenge, and when we talk about challenge, it is not just the challenge from a technical-scientific point of view, but the challenge of reorganizing work practices, a work culture that was already very well established to face a demand that we already imagined that would be overwhelming. (TS25)*

*We gradually incorporated the use of PPE, because we didn’t have this practice (…) establish this flowchart that went all out, the doctor leaving his office and coming here. It was all very scary, but we kept going, we got adapted to it, we established the flowchart that worked, the doctors and nurses who were at the front didn’t get sick. (TS18)*

### Incorporation and Management of the Instruments Application

In this category, the workers’ statements about the incorporation of instruments that were introduced during their work practice to help in the service to users are identified, but such incorporation and use were not followed by relative safety based on scientific evidence, consensus and training, or even on previous experiences.

*The pink code protocol was adopted, some units already mentioned this type of instrument (…), when the patient arrived with some suspicion we already notified him as a pink code patient. (TS12)*

*Basically, the work was of designing the flow, identifying possible bottlenecks in this flow, daily reassessing how it was developed the day before, if there were any slowdowns, anything stuck, anything that required more professionals that could give greater flow to people’s circulation, and involving all professionals, doctors and nurses, technicians, reception, health agents and the Expanded Family Health Center that created the essential tool to follow up. (TS25)*

*So, the protocol is something like that, very personal, varies from doctor to doctor, I never used chloroquine in any of my patients, even when working in my private office (…) we kept the prescription and out of nowhere the Ministry directs us to cancel, for example, Ibuprofen. (TS27)*

*I think that as we effectively worked with the flows for respiratory symptoms, spontaneous demand flows, working with the technical standard, we took all the instruments, we took what was in the literature and we designed it based on our reality. So, I think the operation of respiratory care worked very well. (TS25)*

The use of flows during the development of activities in the crisis, especially at the entrance to the services, was part of these workers’ daily routine. Such standards were constantly put to the test, mainly because they are behaviors still in the validation process. The experience was built as new knowledge emerged, in the decisions and solutions of concrete work problems.

One can see the strategies and actions used by professionals in their work, the constant concern to find ways to work effectively, even with the adversities and limitations generated by the pandemic. This attribution of work, which only experiences are able to provide, is not portrayed in institutional standards and protocols.

*Strategies were built daily for each situation that arose, we created a strategy for each day. So sometimes you had a more severe patient, another more stable, and the strategies were created according to demand, every day a different strategy was created. (TS1)*

*I was already getting tired of having to read different regulations, which were changing every two days, there was a day when you started to have doubts. So we created a group of physicians who were involved. The rule was always to share the doubt and communication was very valuable; all forms of communication were valid [emphasis on WhatsApp groups]; I might not be able to be face to face, but I would call and send messages and we would always find a solution. It was a time of many challenges. (TS30)*

### Professional Experiences and Tactics: Changing Roles, Attitudes, and Relationships

As a striking feature, reports emerged expressing the effects triggered by the pandemic on the worker himself and on each one’s work functioning within the collective work. In everyday situations, such experiences were attributed to attitudinal and relational changes in the face of the challenge of facing the new and the urgent, which leave marks beyond the current moment.

*So, a lot has changed, we are taking more precautions, we are being more careful, washing our hands, something we didn’t do. I didn’t have this habit of spending all the time sanitizing my hands, doing a procedure and washing my hands. I liked to puncture the patient without gloves. (TS07)*

*It changed the whole routine of all professionals, we used to not wear PPE, even the necessary ones. Not today, everyone arrives and goes straight to a room and puts on a cap, mask, apron, shoe covers, face shield, so it was this routine every day. Today it has already been quit, but we continue using it, it has changed everything radically. (TS08)*

*We weren’t used to using all these parameters that we use today, in fact it was already for us to use (…). So, we had to be already used to it, but, in reality, nobody was used to it. So, when COVID broke out, it was oppressive: it oppressed us because of that, that we had to wear all the PPE. (TS20)*

*So, we had to be updated and filter what was and what was not worth it based on the experience of other colleagues; because there were people who used chloroquine for everybody, even without the ECG and then, it turns out that we don’t have the scientific evidence for such an action. (TS27)*

Ways of carrying out work safely for oneself and others (colleagues and users) reiterated responsibilities and technical rigor on a scale of severity not compared to anything before.

*We had to be more careful with our own safety. Right at the beginning it wasn’t enough, I was infected right at the beginning of the pandemic. When I returned to the unit after 16 days away, I had to be even more careful. (TS24)*

## DISCUSSION

The reports showed that workers have unique discursive ways of explaining their experience in coping with COVID-19, configuring narratives about apparently simple and commonplace work situations, but with great transforming power. The category “reorganization of services and functions”, for example, points to a new look at the organization of work based on everyday life in motion – within antecedent standards, urgent (and sometimes precarious) renormalizations, incorporation and management of ingredients, both new ones and those already constituents of the labor heritage. Activity management presupposes assuming work as an enigmatic reality, hybrid between the visible and the invisible, whose renormalizing result is shown both in the task performed and in the critical evaluation of the antecedent standards, allowing the worker to create alternative reserves^(8)^.

In this scenario, they experience, mediate and act in the most diverse situations. Ergology brought important conceptual supports to interpret workers’ reports about what they did, with what they did, and how they themselves changed while changing their work.

Obviously, there are legitimate uses in recognizing the experiences of workers in facing a pandemic that exposed so much precariousness, especially in terms of work organization, management of services and systems, and evaluation of policies, especially in the context studied. However, one cannot help thinking about those who lived through the extraordinary time and will inherit its legacy.

In the debate about standards and in the dramas of the use of the self, the feeling of emptiness or insufficiency of standards is caught, in a scenario of uncertainties, little clarity, and juxtaposition of directions. At the same time, in the face of situations of coping with the pandemic, workers develop experiences and tactics that configure changes in roles, intra- and extra-team relationships, and ways of acting and expressing themselves.

In this context, work plays a central role in the analysis of disease control strategies^(17)^. The work is carried out in a process in which the product or result responds to a concrete and socially produced purpose, according to human needs^(18)^. Thus, health work is understood as a practice of professionals whose purpose is to develop actions related to the care of patients and/or clients^(19)^.

The preparation of health services and human resources is inexorable in the way it impacts the entire process of coping with a pandemic that, in some scenarios, has contaminated 80% of the population. The high transmissibility of the virus, associated with inadequate protection, overwork, and health workers exhaustion generated greater frustration in the latter for not being able to meet so much demand with quality^(20)^.

As work involves social requirements, imposed purposes and regulated tasks, no matter how intensely they are prescribed, they are performed by unique individuals in variable contexts, which results in each activity being unique, although it may be similar^(12)^.

In the ergological approach, work is not simply the execution of tasks or compliance with routines and procedures. Every work activity is a drama of the *use of self*, traversed by antecedent standards and by the worker’s need to supply and act in their activities, expressing their values and their singularities^(21)^.

The normative debate presented here leads to other ways of seeing things, as well as other visions of living and acting together, from the more macro aspects of possibilities, such as the formulation of flows, to behavioral changes, such as the use of PPE. In this process, there is a permanent production of renormalization, being part of a constant dynamic in the work organization process, which is not limited to the task, it goes far beyond, it refers to the act of a person who creates and recreates in carrying out work activity^(21)^.

In the context studied, health workers seek alternatives to organize their work and manage their own activity in an environment of great exposure to risk, emphasizing the rapid establishment of a differentiated service flow for patients with suspected COVID-19 and the effective use of PPE as a way of protection. The risk was recognized at every minute, relentlessly and with no relaxation, in the face of what was already known, transformed by new contingencies and severity – illness and death. Daily life made the use of oneself at work problematic, insofar as the safety of each person required attention to the smallest details, highlighting the relationship between small actions and big fears. From these workers, immediate decision- making was required, influenced by standards, processes, and public policies, as well as technical skills and legal limits of professional practice, in movements under flexibility margins that were never completely predetermined and normalized^(9)^.

The debate on standards involves tensions and agreements between precepts imposed and established in action, as a result of this necessary implication in work, of never being defined only by the normative imposition of rules and purposes of work managers. It is the meaning given to the work itself that determines the worker’s involvement in the role played. The experiences of the professionals assessed in the study showed not only the reinvention of the activity in a scenario where previous standards were put to the test at great speed, but also the reconfiguration of skills. For this, elements such as training and professional experience may have played an important role (most graduates and with 7 years or more of experience), although the intensity of the dramas of use of the self hinders finding more or less preponderant elements. The competence built in the activity is done in the synthesis of heterogeneous and interrelated ingredients – from the appropriation of knowledge and antecedent standards, the apprehension of the situation as an experience or encounter and, finally, the debate of values that summons the worker to complex negotiations, which does not deny the weight of what the environment makes available to the person as a space to expand their potential^(22)^. Closely related to this environmental component is the lack of adequate conditions to carry out the work, since workers are impacted by difficulties with inadequate materials, equipment, and structures to carry out their work, as well as insufficient conditions of means of protection and precariousness of the workforce^(23)^. This has been exhaustively described in different scenarios around the world, pointing to the importance of structural, personnel, and support conditions for the serious consequences for the mental health of workers who face the pandemic^(24,25)^.

In the context studied, unacceptable consequences were produced with the collapse of the health system in early 2021, with loss of lives due to the precariousness of basic conditions of treatment and preparation of services. These situations intensify stress and moral suffering for workers^(26)^ who do not see their conditions of work ensured, with safety, dignity, or effectiveness.

In the daily work of health workers in the Health Care Network (*RAS*) in Manaus, the drama of *use of self* is visible, with the peculiarities in the service imposed by the urgency of responses in the pandemic being highlighted, which demanded the workers’ agility, skill, and speed. The study revealed strategies and actions used by professionals in their work, seeking to find a way of working, despite the limitations of the physical structure, material, and constant changes in protocols. Volatility and inconstancy mark even what was once solid and reliable, such as protocols, to the point of being identified as a resource and product of individual practice – “each one has their own”. The contradictory of an expression that portrays the insufficiency of antecedent standards and the acceptance of anything that took its place, in the individualization of the appropriation of knowledge and technology. The incorporation and management of the application of instruments showed, paradoxically, simple and agile responses, alternative and peripheral means of communication (such as exchanges of opinions via WhatsApp groups), outside the official information circuits. This finding may be related to the digital communication architecture used by the federal government, to operate borders of ignorance within science itself^(27)^. In fact, the tactics of communication used in the different management levels of the Brazilian Public Health System (*SUS*) did not adequately address the themes of surveillance, assistance, health promotion and education, being guided by a utilitarian, centralizing, and functional model (transmission and not emancipation)^(28)^. The consequences of denialism and authoritarianism in communication management lead to loss of transparency, risk analysis, suitability, and punctuality^(29)^.

Added to insecurity in action are exhausting routines and care that can become painful and not very successful. In this routine, professional experiences and strategies were reconfigured, relativizing and modifying roles, attitudes, and work relationships, even giving a new meaning to self-protection at work.

Finally, interesting examples of health care reorganization modalities show the protagonism of workers directly involved in care actions for people suspected of COVID-19, in the creation of different strategies to provide services to users and even reduce the level of spread of the disease^(30)^, what was present in the experiences reported by the participants.

The results confirmed that, in the context of the current crisis, the experience and knowledge that have been developed in the field of health services make their work processes and the construction of their collective heritage undergo major transformations^(30)^.

In this regard, the need to consider the workers’ health is also highlighted, emphasizing the importance of supporting workers by protecting their mental health, through training and measures to ensure safe work and occupational safety, allowing fair treatment for people affected by COVID-19 and other illnesses^(31)^.

## CONCLUSIONS

By exploring workers’ narratives about coping with the COVID-19 pandemic in Manaus *RAS* services, the study highlighted a work organization dynamized by urgency and risk. The severity and intensity of the crisis experienced in the municipality studied point to the importance of work management based on a theoretical basis that places professional experience at the center of reflection and of the construction of new and edifying alternatives for health work. When discussing past standards and comparing his/her knowledge, experiences and values with the new demands, the worker modifies the environment, flows, and conducts as needed, facing the lack of safety, conditions and solidity of the technical bases of work.

Dialoguing with ergology, it is important to recognize that in health work, quick decision-making helps workers to act in the best possible way, renormalizing routines and providing care. The debate on standards and the dramas of the use of the self enhance the understanding of the work process of the professionals involved. The analytical axis based on these concepts did not take them as an *a priori* category, but as a conceptual background, procedurally conformed by the three empirical categories, focusing on work reorganization, on the incorporated instruments, and on the changes about themselves and their relationships, without any elements overlapping or being able to be designed in isolation.

Therefore, the results of this study provide important information about the complexity of work management in coping with the pandemic in Manaus, allowing reflections to be triggered on the multiple objective and subjective dimensions that involve the built experiences. Some findings show aspects already widely portrayed in the pandemic context, such as the reorganization of work environments, reduction in workload, guidance and encouragement for the use of PPE, and mental health care for these workers. However, the ergological framework helps to broaden the scope of reflection, integrating new elements for a critical and transforming view of the work that is intended to be resolutive, safe, and consistent with the purposes of excellence of the care offered and the professional practice that is full of values.
